# Influence of leptin administration to pregnant mice on fetal gene expression and adaptation to sweet and fatty food in adult offspring of different sexes

**DOI:** 10.18699/vjgb-24-33

**Published:** 2024-06

**Authors:** E.I. Denisova, E.N. Makarova

**Affiliations:** Institute of Cytology and Genetics of the Siberian Branch of the Russian Academy of Sciences, Novosibirsk, Russia; Institute of Cytology and Genetics of the Siberian Branch of the Russian Academy of Sciences, Novosibirsk, Russia

**Keywords:** adaptation to high-calorie food, developmental programming, leptin, mice, pregnancy, адаптация к высококалорийной пище, программирование развития, лептин, мыши, беременность

## Abstract

Elevated leptin in pregnant mice improves metabolism in offspring fed high-calorie diet and its influence may be sex-specific. Molecular mechanisms mediating leptin programming action are unknown. We aimed to investigate programming actions of maternal leptin on the signaling function of the placenta and fetal liver and on adaptation to high-calorie diet in male and female offspring. Female C57BL/6J mice received leptin injections in mid-pregnancy. Gene expression was assessed in placentas and in the fetal brain and liver at the end of pregnancy. Metabolic parameters and gene expression in the liver, brown fat and hypothalamus were assessed in adult male and female offspring that had consumed sweet and fatty diet (SFD: chow, lard, sweet biscuits) for 2 weeks. Females had lower blood levels of leptin, glucose, triglycerides and cholesterol than males. Consuming SFD, females had increased Ucp1 expression in brown fat, while males had accumulated fat, decreased blood triglycerides and liver Fasn expression. Leptin administration to mothers increased Igf1 and Dnmt3b expression in fetal liver, decreased post-weaning growth rate, and increased hypothalamic Crh expression in response to SFD in both sexes. Only in male offspring this administration decreased expression of Fasn and Gck in the mature liver, increased fat mass, blood levels of glucose, triglycerides and cholesterol and Dmnt3a expression in the fetal liver. The results suggest that the influence of maternal leptin on the expression of genes encoding growth factors and DNA methyltransferases in the fetal liver may mediate its programming effect on offspring metabolic phenotypes.

## Abstract (ru)

Повышенный уровень лептина в период беременности у самок мышей оказывает благоприятное действие на метаболические показатели их зрелого потомства при потреблении последним высококалорийной пищи, и это влияние может зависеть от пола. Молекулярные механизмы, опосредующие программирующее действие лептина, неизвестны. Целью представленной работы было изучение программирующего действия материнского лептина на сигнальную функцию плацент и печени плодов, а также на адаптацию к высококалорийной диете у потомства в зависимости от пола. Самкам мышей линии C57BL/6J вводили лептин в середине беременности. В конце беременности в плацентах, мозге и печени плодов оценивали экспрессию генов. У взрослого потомства обоего пола оценивали метаболические показатели и экспрессию генов в печени, буром жире и гипоталамусе после двухнедельного потребления стандартной либо сладко-жирной диеты (СЖД: гранулы стандартного корма, сало, сладкое печенье). У самок наблюдался более низкий уровень лептина, глюкозы, триглицеридов и холестерина в крови, чем у самцов. Потребление СЖД увеличивало экспрессию гена Ucp1 в буром жире у самок, тогда как у самцов накапливался жир, снижались уровень триглицеридов в крови и экспрессия гена Fasn в печени. Введение лептина матерям увеличивало экспрессию генов Igf1 и Dnmt3b в печени плодов, снижало скорость роста после отъема от матери и повышало экспрессию Crh в гипоталамусе в ответ на СЖД у взрослых потомков обоих полов. Только у самцов введение лептина матерям снижало экспрессию генов Fasn и Gck в печени, увеличивало жировую массу, уровни глюкозы, триглицеридов и холестерина в крови, а также экспрессию гена Dmnt3a в печени плодов. Полученные результаты позволяют предположить, что влияние материнского лептина на экспрессию генов, кодирующих факторы роста и ДНК- метилтрансферазы в печени плодов, может опосредовать его программирующий эффект на метаболический фенотип потомства

## Introduction

Obesity and related metabolic diseases are one of the major problems in modern medicine. The potentiating effect of maternal obesity on the development of obesity in the offspring is considered as one of the reasons for the widespread prevalence of obesity (Shrestha et al., 2020; Schoonejans et al., 2021). In this regard, the study of the possible mechanisms responsible for mediating the effects of early-life environment on susceptibility to obesity later in life is of particular relevance.

The adipocyte hormone leptin can have a programming effect on the development of offspring. It was shown in laboratory models that elevated blood levels of leptin in pregnant females, whether caused by genetic disorders or leptin administration, may have a beneficial effect on glucose metabolism and obesity in offspring fed a high-calorie diet (Stocker, Cawthorne, 2008; Pollock et al., 2015; Talton et al., 2016; Denisova et al., 2021). It was also shown that the programming effects of maternal leptin can be different in offspring of different sexes (Nilsson et al., 2003; Makarova et al., 2013). The study of the molecular and physiological mechanisms that mediate the programming effect of leptin may contribute to the elaboration of methods for correcting individual development to reduce the risk of metabolic disease

In most cases, the development of obesity is promoted by the consumption of high-calorie sweet and fatty food. Adaptation to the consumption of this type of food is expressed in a decrease in the amount of food consumed, storage of excess energy in adipose tissue, and an increase in energy expenditure (Duca et al., 2014). These adaptive responses are associated with changes in the expression of orexigenic and anorexigenic neuropeptides in the hypothalamus (Cone, 2005), activation of thermogenesis in brown adipose tissue (Even, 2011), and changes in the activity of enzymes related to glucose and lipid metabolism in the liver and other organs (Akieda-Asai et al., 2013). Ability to adapt to the consumption of high-calorie foods may affect the rate and degree of obesity development. However, the effect of maternal leptin on adaptation to sweet and fatty foods has not yet been investigated

The programming effect of maternal leptin on the development of offspring can be mediated via epigenetic modifications, including methylation of regulatory regions of genes and changes in the expression of signaling factors that affect the growth and maturation of organs and tissues in fetuses (Reynolds et al., 2017). Insulin-like growth factors 1 and 2 (IGF1, IGF2) play a significant role in the somatic development of the fetus (Petry et al., 2010; Xiagedeer et al., 2020; Hattori et al., 2021). These factors are synthesized and secreted into the blood of the fetus by both placenta and fetal liver (Nawathe et al., 2016). The effect of maternal leptin on the signaling function of the placenta and fetal liver has not yet been studied.

The aim of this study is to investigate the effect of increased leptin levels in pregnant females on the signaling function of the placenta and fetal liver and on the adaptation to the consumption of high-calorie sweet and fatty foods in mature offspring of different sexes in mice.

## Materials and methods

Animals and experimental design. The study was conducted according to the guidelines of the Declaration of Helsinki and approved by the Independent Ethics Committee of the Institute of Cytology and Genetics, Siberian Branch, Russian Academy of Sciences (protocol number 76, 07.04.2021).

Experiments were conducted with С57BL/6J mice housed at the vivarium of the Institute of Cytology and Genetics, Novosibirsk, Russia. The animals were kept at a 12-h daylight cycle with free access to water and standard chow for the conventional maintenance and breeding of rodents (BioPro Company, Novosibirsk, Russia). Mature females were mated to males of the same strain. Mating was confirmed by the presence of a copulation plug. The appearance of the plug signified day 0 of pregnancy. The females were administered 0.2 mg/kg of recombinant murine leptin (Peprotech, United Kingdom) or the same volume of normal saline on days 11, 12, and 13 of pregnancy. The injections were done subcutaneously in the shoulder area. It has been shown that during this period, sexual differentiation begins in fetuses (Hacker et al., 1995) and there is a peak in the formation of hypothalamic neurons that regulate energy intake and expenditure (Ishii, Bouret, 2012). As we showed earlier, the food intake of females reduces in response to leptin administration, and the offspring demonstrate sensitivity to its programming effect during this period of pregnancy (Denisova et al., 2021).

To study the effect of leptin administration on the fetal growth and expression of genes in fetuses and placentas, 6 leptin-treated and 6 control females were sacrificed at the pregnancy day (PD) 18 by displacement of the cervical vertebrae, fetuses and placentas were removed and weighed. Samples of placentas and fetal liver and brain were placed in liquid nitrogen. To measure gene expression, two tissue samples of the placentas and fetuses of each sex were selected from each litter and combined in equal representation, taking into account the RNA concentration after RNA isolation.

In another group, the mated females were monitored to record parturition and the number of pups, and the day of delivery was designated as postpartum day (PPD) 0. Females with a litter of less than 6 pups did not participate in the further experiment. If there were more than 7 pups in the litter, it was adjusted to 7 on PPD 0. There were 9 leptin-treated litters and 8 control litters. The females and pups were weighed on PPDs 0, 7, 14, 21, and 28. The offspring were weaned from their mothers at PPD 28.

To assess the effect of maternal leptin on the metabolic parameters of mature offspring, two males and two females from each litter were housed individually after weaning. At the age of 10 weeks, some of the offspring begun to receive a sweet and fatty diet (SFD): sweet butter cookies and lard were added to standard chow, and the other part of the animals remained on standard diet (SD). There were 8 experimental groups with 6–7 animals in each group: males and females consuming SFD and males and females consuming SD born to control mothers and males and females consuming SFD and males and females consuming SD born to leptin-treated mothers. The weight of standard chow, fat and cookies eaten per week was measured, and energy intake was calculated (lard – 8 kcal/g, cookies – 4.58 kcal/g, and standard chow – 3 kcal/g). The total amount of energy consumed was calculated and related to body weight.

After 2 weeks of SFD eating, the animals were decapitated, the weight of the liver, interscapular brown fat, and subcutaneous and intraperitoneal fat were measured. To assess the effect of leptin on blood biochemical parameters and gene expression, blood samples were collected, liver, muscle, brown fat and hypothalamus samples were placed in liquid nitrogen and then stored at –80 °C.

Plasma assays. Concentrations of leptin and FGF21 were measured using Mouse Leptin ELISA Kit (EMD Millipore, St. Charles, MO, USA) and Quantikine® ELISA Mouse/ Rat FGF-21 Immunoassay (R&D Systems, Minneapolis,USA).

Concentrations of glucose, triglycerides, and cholesterol were measured colorimetrically using Fluitest GLU, Fluitest TG, and Fluitest CHOL (Analyticon® Biotechnologies AG Am Mühlenberg 10, 35,104 Lichtenfels, Germany), respectively

Relative quantitative real-time PCR. Gene expression was measured using relative quantitative real-time PCR. Total RNA was isolated from tissue samples using the ExtractRNA kit (Evrogen, Moscow, Russia) according to the manufacturer’s instructions. First-strand cDNA was synthesized using Moloney murine leukemia virus (MMLV) reverse transcriptase (Evrogen, Moscow, Russia) and oligo(dT) as a primer. TaqMan gene expression assays (Thermo Fisher Scientific, Waltham, MA USA) indicated in Table 1 were used for relative quantitative real-time PCR with β-actin (Actb) and cyclophilin (Ppia) as an endogenous control

**Table 1.**
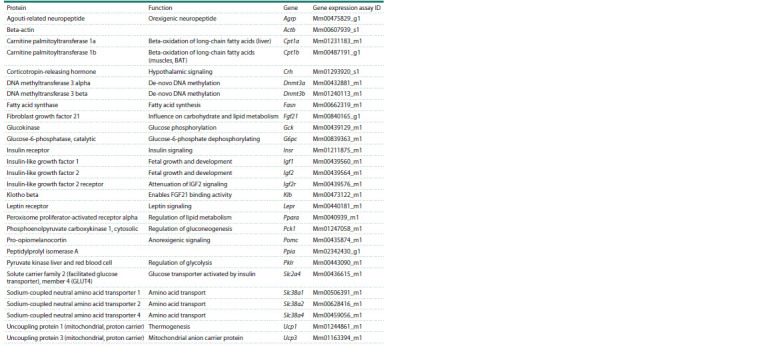
TaqMan Gene Expression Assays used for relative quantitative real-time PCR

Sequence amplification and fluorescence detection were performed on a QuantStudio™ system. Relative quantification was performed by the comparative threshold cycle (CT) method.

Statistical analyses. Data were analyzed with the STATISTICA 10.0 program. Descriptive statistic was used to determine means and standard error (SE) of the mean. Data on body weight and food intake were analyzed using Repeated Measures ANOVA with factors “maternal treatment” (administration of leptin or saline), “sex”, and “age” (from 4 to 10 weeks) for offspring when kept on a standard diet. When kept on a sweet and fatty diet, data on energy intake were analyzed using Repeated Measures ANOVA with factors “diet” (SD and SFD), “maternal treatment” and “age” (from 10 to 12 weeks) and data on weight gain were analyzed using two-way ANOVA with factors “diet” and “maternal treatment” separately for male and female offspring. Morphometric, metabolic and hormonal parameters and gene expression were analyzed initially by three-way ANOVA with factors “maternal treatment,” “diet,” and “sex” and then separately by two-way ANOVA in offspring consuming SD or SFD with factors “sex” and “maternal treatment,” or in males and females with factors “maternal treatment” and “diet”. To identify the effect of leptin administration on the weight of fetuses and placentas and gene expression in fetuses and placentas, two-way ANOVA was used with factors “sex” and “maternal treatment”. To assess intergroup differences, post hoc Newman–Keuls test was used. The comparisons between single parameters were performed with a two-tailed Student’s t-test. The results on the graphs are presented as mean ± SE. Significance was determined as p < 0.05.

## Results

The effect of leptin administration to pregnant mice on body weight and energy intake in offspring of different sexes when kept on SD

The administration of leptin to pregnant females had no effect on body weight (BW) of the offspring at birth and during the period of maternal care (PPDs 1–28); no sex differences in BW were observed during this period either.

**Fig. 1.**
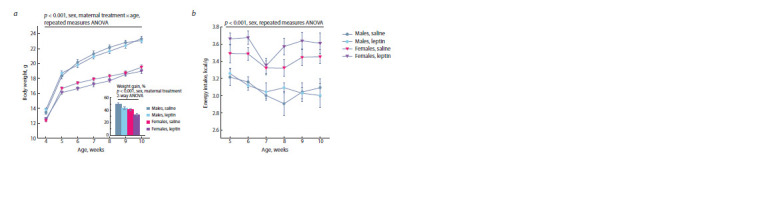
The effect of leptin administration to female mice at 11–13 days of pregnancy on weight gain during the first two weeks after weaning (a) and body weight (a) and weekly energy intake related to body weight (b) at the age of 4–10 weeks in offspring of different sexes when consuming a standard diet Data are means ± SE from 12–14 animals in every group. Weight gain was calculated as the difference in weight in the first two weeks after weaning divided by weight at the weaning and expressed as a percentage.

After weaning, males as compared to females had a higher growth rate and were significantly heavier (Fig. 1a). The administration of leptin to mothers affected the dynamics of weight gain in both males and females; it reduced the growth rate of the offspring in the first two weeks after weaning (Fig. 1a). Females consumed more energy per unit of body weight than males (Fig. 1b), leptin administration to mothers had no effect on offspring energy intake

The effect of leptin administration to pregnant mice on energy intake and body weight in offspring of different sexes when kept on SFD

**Fig. 2.**
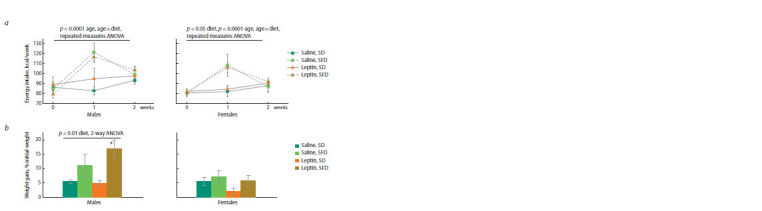
Influence of leptin administration to pregnant mice on energy intake (a) and weight gain (b) in male and female offspring consuming standard or sweet and fatty diet. Data are means ± SE from 6–7 animals in every group. * p < 0.05, SFD vs. SD, post hoc Newman–Keuls test.

Energy consumed with SFD changed dramatically in the course of the experiment: it increased sharply in comparison with the control in the first week, and returned to normal in the second week in mice of both sexes (Fig. 2a). The leptin administration to mothers had no effect on the dynamics of energy intake with SFD in the offspring. At the same time, there were sex differences in BW changes resulting from SFD consumption ( p < 0.05, “sex” × “diet”, 3-way ANOVA): SFD did not affect weight gain in females, and increased weight gain in males, especially in the offspring of leptin-treated mothers (Fig. 2b).

Influence of leptin administration to pregnant mice on the metabolic characteristics in offspring of different sexes when kept on SD or SFD

When offspring consumed SD, sex differences were observed in many morphometric and biochemical parameters. Two-way ANOVA with factors “sex” and “maternal treatment” showed that females compared with males had decreased absolute and relative weights of brown adipose tissue (BAT) ( p < 0.001, absolute, p < 0.05, relative, “sex”) and intraperitoneal white adipose tissue (WAT) ( p < 0.01, absolute, p <0.05, relative, “sex”) (Table 2), and lowered levels of glucose ( p < 0.05, “sex”), cholesterol ( p < 0.01, “sex”), triglycerides ( p < 0.001, “sex”) and leptin ( p < 0.05, “sex”) in the blood (Table 3). Leptin administration to pregnant mothers was associated with an increase in blood triglyceride levels ( p < 0.05, “maternal treatment”), and this increase reached statistically significant values in male offspring ( p < 0.05, post hoc Newman–Keuls test)

**Table 2.**
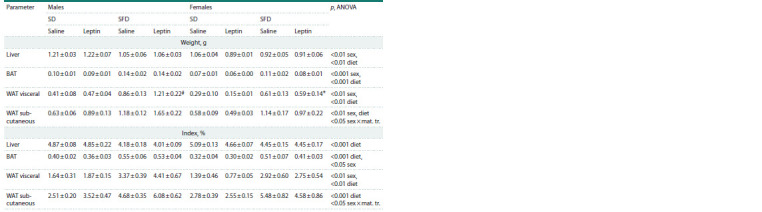
Influence of leptin administration to pregnant mice on the absolute and relative weight of the liver, BAT, and visceral and subcutaneous WAT in male and female offspring consuming SD or SFD Note. Data are means ± SE from 6–7 animals in every group. Data were analyzed using three-way ANOVA with factors “sex”, “diet”, and “maternal treatment” (mat. tr.). * p < 0.05 females vs. males, # p < 0.05 SFD vs. SD, post hoc Newman–Keuls test.

**Table 3.**
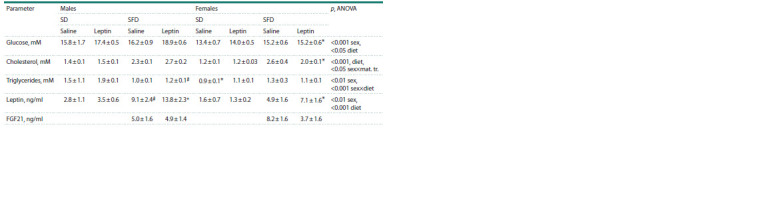
Influence of leptin administration to pregnant mice on hormonal and metabolic characteristics in male and female offspring consuming SD or FSD Note. Data are means ± SE from 6–7 animals in every group. Data were analyzed by three-way ANOVA with factors “sex”, “diet” and “maternal treatment” (mat. tr.). * p < 0.05 females vs. males, # p < 0.05 SFD vs. SD; + p < 0.05 males, leptin vs. saline, post hoc Newman–Keuls test.

A two-week intake of SFD reduced the absolute and relative weight of the liver, increased the absolute and relative weight of BAT, as well as visceral and subcutaneous WAT, and increased the blood levels of glucose, cholesterol and leptin in both males and females (Tables 2, 3). Only the change in blood triglyceride levels in response to the consumption of SFD depended on sex: triglyceride levels decreased in males and did not change in females (Table 3). At the same time, in females, the mass of visceral WAT and the concentration of glucose, cholesterol, and leptin in the blood were lower than in males, regardless of the diet consumed (Tables 2, 3). Leptin administration to mothers had a sex-specific effect on the mass of subcutaneous WAT and blood glucose, cholesterol, and triglyceride levels. When the effect of maternal leptin was analyzed separately in males and females (two-way ANOVA with factors “diet” and “maternal treatment”), it was observed only in males. Regardless of the diet, male offspring of leptintreated mothers had more subcutaneous fat mass ( p < 0.05, “maternal treatment”) and elevated blood levels of glucose ( p < 0.05, “maternal treatment”), triglycerides ( p <0.05, “maternal treatment”) and cholesterol (at the trend level, p < 0.07, “maternal treatment”) than males born to control mothers.

Influence of leptin administration to pregnant mice on gene expression in the liver, BAT and muscles in male and female offspring consuming SFD or SD

**Fig. 3.**
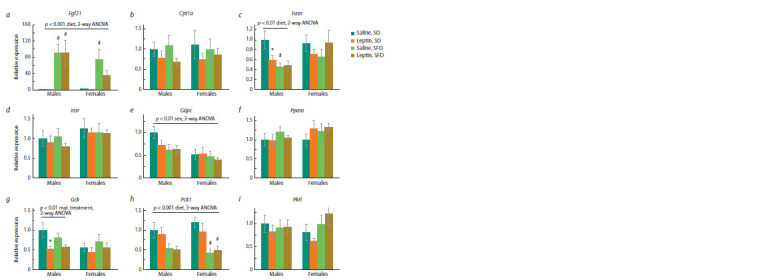
Influence of leptin administration to pregnant mice on liver gene expression in male and female offspring consuming SFD or SD * p < 0.05 SD, males, leptin vs. saline; # p < 0.05 SFD vs. SD, post hoc Newman–Keuls test. Data are means ± SE from 6–7 animals in every group.

**Fig. 4.**
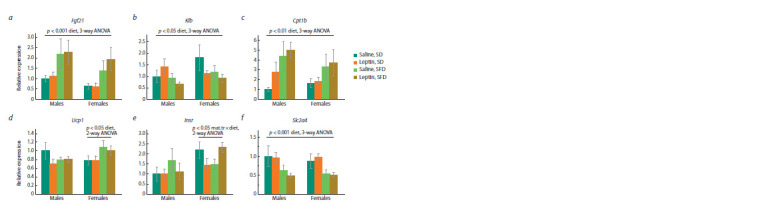
Influence of leptin administration to pregnant mice on gene expression in BAT in male and female offspring consuming SFD or SD. Data are means ± SE from 6–7 animals in every group.

When mice were kept on a standard diet, sex differences were observed in the expression of some of the studied genes in the liver and brown fat. In the liver, the mRNA level of glucose- 6-phosphatase (G6pc) in females was lower than in males ( p <0.05, “sex”, two-way ANOVA, SD, Fig. 3e). In BAT, the FGF21 mRNA level in females was lower than in males, and the level of insulin receptor mRNA was higher ( p < 0.05, “sex”, for both cases, two-way ANOVA, SD, Fig. 4a, e). Leptin administration to mothers reduced the expression of Fasn ( p < 0.05, “maternal treatment”, two-way ANOVA, SD, Fig. 3c) and Gck ( p < 0.05, “maternal treatment”, two-way ANOVA, SD, Fig. 3g) in the liver on a standard diet, and this decrease was more pronounced in males, reaching statistically significant values in them (Fig. 3c, g).

In the liver, SFD consumption resulted in activation of Fgf21 gene expression and inhibition of Pck1 gene expression in both males and females (Fig. 3a, h), and inhibition of Fasn gene expression only in males ( p < 0.01, “diet”, twoway ANOVA, males, Fig. 3c). At the same time, in males,leptin administration to mothers changed the response of the Fasn gene to SFD consumption: in the offspring of control mothers, Fasn gene expression significantly decreased, while in the offspring of leptin-treated mothers, it did not change (Fig. 3c). Leptin administration to mothers also had a sexspecific effect on the expression of the glucokinase gene in the liver – it decreased in males regardless of the diet and did not significantly change in females (Fig. 3g).

In BAT, SFD consumption increased Fgf21 and Cpt1 gene expression (Fig. 4a, c), decreased Slc2a4 gene expression (Fig. 4 f ), had a down-regulating effect on Klb expression (Fig. 4b) in mice of both sexes, and increased Ucp1 gene expression only in females (Fig. 4d). Leptin administration to mothers had no effect on the expression of the studied genes in BAT.

In the muscles, the expression of genes related to insulin sensitivity (Slc2a4, Insr) and β-oxidation (Cpt1b, Ucp3) were studied. The expression of these genes did not depend on sex and diet, and leptin administration to mothers had no effect on the expression of these genes.

Influence of leptin administration to pregnant mice on hypothalamic gene expression in male and female offspring consuming SFD or SD

**Fig. 5.**
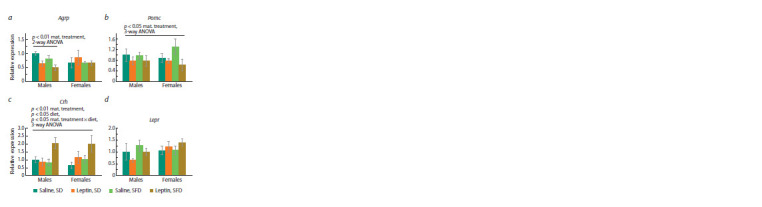
Influence of leptin administration to pregnant mice on gene expression in hypothalamus in male and female offspring consuming SFD or SD. Data are means ± SE from 6–7 animals in every group.

When kept on SD, males and females did not differ in the expression of the studied genes in the hypothalamus. Leptin administration to mothers had a down-regulating effect on Pomc gene expression regardless of animal sex and diet (Fig. 5b), reduced Agrp gene expression only in males (Fig. 5a) on both SD and SFD, and altered the response of the Crh gene to SFD intake. In mice of both sexes born to leptin-treated mothers, the expression of the Crh gene increased when SFD was consumed, while in the offspring of control females it did not change (Fig. 5c). Expression of Agrp, Pomc, and Lepr did not change in response to SFD consumption.

The results presented suggest that maternal leptin has a programming effect on the metabolic phenotype of the offspring, including influence on the central mechanisms supporting energy homeostasis, and gene expression in the liver and brown fat, and males are more sensitive to the programming action of maternal leptin.

Influence of leptin administration to pregnant mice on the weight of placentas and fetuses in offspring of different sexes

**Fig. 6.**
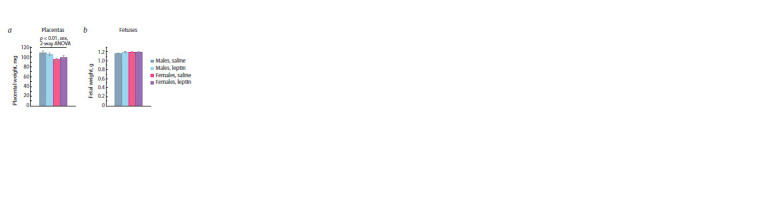
The effect of leptin administration to female mice at 11–13 days of pregnancy on the weight of placentas (a) and fetuses (b) of different sex at the end of pregnancy (PD 18). Data are means ± SE from 32 male and 20 female offspring of control mothers and 29 male and 24 female offspring of leptin-treated mothers.

Leptin administration to mothers at mid-pregnancy did not affect fetus viability: control and leptin-treated mothers did not differ in litter size (8.7 ± 0.2, n = 6, control mothers, and 9.0 ± 0.2, n = 6, leptin-treated mothers). At the end of the embryonic period, male and female fetuses did not differ in weight, and leptin administration to mothers did not have a delayed effect on fetal weight (Fig. 6b). Male placentas weighed more than female placentas (Fig. 6a). Leptin administration to mothers had no effect on placental or fetal weight.

Influence of leptin administration to pregnant mice on gene expression in placentas, and in the brain and liver of fetuses of different sexes

**Fig. 7.**
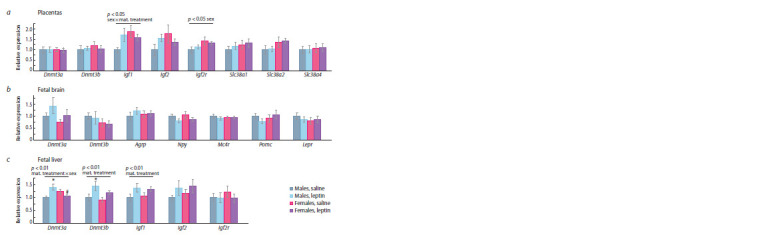
Influence of leptin administration to female mice at the days 11–13 of pregnancy on gene expression in placentas (a), fetal brain (b) and fetal liver (c) in male and female fetuses at the end of pregnancy (PD 18). Data are means ± SE from 6 samples in every group. * p < 0.05, male fetuses, leptin vs. saline; # p < 0.05, leptin, females vs. males, post hoc Newman–Keuls test.

In the control, female fetus placentas differed from male fetus placentas by increased expression of the Igf1 gene ( p < 0.05, Student’s t-test). Administration of leptin to pregnant mice affected the placental expression of this gene differently in male and female fetuses ( p < 0.05, “sex” × “maternal treatment”, two-way ANOVA): it increased Igf1 expression in male placentas and decreased in female placentas (Fig. 7a). As a result, the sex differences in Igf1 expression observed in the control group disappeared when leptin was administered to mothers.

The expression of the Igf2r gene and, at the level of a trend, the Slc38a2 (SNAT2) gene ( p = 0.054, two-way ANOVA) in placentas (Fig. 7a) depended on the sex of the fetuses: it was higher in females than in males, and leptin administration to pregnant females had no effect on the expression of these genes.

Sex differences in the expression of the genes studied in the fetal brain and the effect of leptin administration to pregnant females on the expression of these genes were not found (Fig. 7b).

Sex differences in the expression of the genes studied in the liver were not found. Leptin administration to pregnant females had an up-regulating effect on the liver expression of the Igf1 and Dnmt3b genes in the fetuses of both sexes and a multidirectional effect (up-regulating in males and down-regulating in females) on the liver expression of the Dnmt3a gene (Fig. 7c). As a result, Dnmt3a gene expression in male fetuses was higher than in female fetuses after leptin administration to mothers.

Thus, administration of leptin to females during pregnancy has a delayed effect on the expression of genes encoding growth factors and DNA methyltransferases in the fetal liver

## Discussion

In the present work, we assessed the effect of maternal leptin on adaptation to high-calorie food in adult offspring, as well as on the signaling function of placentas and fetal liver depending on offspring sex. Sex has a significant effect on obesity-induced metabolic alterations (Hwang et al., 2010), and, in addition, there is sexual dimorphism in the response of offspring to maternal influences not only in the postnatal period of life, but also in fetuses and placentas (Dearden et al., 2018; Yu et al., 2021). It suggests that the programming effect of maternal leptin may be sex-specific

Male and female offspring differed in metabolic characteristics consuming SD and in response to SFD intake. Compared to males, females had reduced fat mass and reduced blood glucose, cholesterol, and leptin concentrations regardless of the diet consumed, which is consistent with the results of other authors (Freire-Regatillo et al., 2020). SFD consumption was accompanied by an increase in the intake of energy in the offspring of both sexes, but the utilization of this excess energy depended on the sex. In males, when switching to SFD, the mass of white fat increased, the expression of the Fasn gene encoding the enzyme for the synthesis of fatty acids decreased in the liver, and the level of triglycerides in the blood decreased. These results are consistent with data obtained in other studies on male mice (Voigt et al., 2013; Casimiro et al., 2021; Kakall et al., 2021) and suggest that in males, excessive consumption of fat at the initial stages of fatty food eating inhibits lipogenesis in the liver and enhances lipid uptake by tissues and lipid storage in adipose tissue. In females, the mass of adipose tissue, liver expression of Fasn, and blood triglyceride level did not change in response to SFD but the expression of the Ucp1 gene in BAT increased, which indicates an increase in thermogenesis and energy dissipation in the form of heat. Thus, males and females demonstrate different adaptive strategies in relation to excess energy intake with SFD.

In other respects, the hormonal and metabolic changes induced by the intake of SFD were similar in males and females and were aimed at reducing food intake, lowering blood glucose levels, and activating fat utilization. In offspring of both sexes, energy intake declined to normal levels in the second week of SFD intake, which may be due to an increase in leptin levels, because leptin reduces food intake (Morton, 2007). In both males and females, the mass of BAT increased and BAT expression of the Cpt1 gene increased and that of the Slc2a4 gene (GLUT4) decreased, which points to intensification of lipid utilization. In addition, liver mass decreased and liver Pck1 gene expression decreased, which indicates the suppression of gluconeogenesis. The expression of the Fgf21 gene increased in the liver and brown fat. This hormone increases insulin sensitivity, activates fat oxidation, and influences food choice, increasing the propensity to consume a balanced diet (Flippo, Potthoff, 2021). These results are consistent with data obtained by other authors. It has been shown in mice and rats that the initial stages of adaptation to the consumption of a high-calorie diet are characterized by an increase in energy expenditure, an increase in the level of leptin in the blood, an increase in the mass of brown fat, UCP1 protein expression and fatty acid oxidation in brown fat, an increase in fat utilization, a decrease in liver weight, a decrease in the expression of the Slc2a4 gene (GLUT 4) in adipocytes (So et al., 2011; Andrich et al., 2018; Kakall et al., 2021).

Leptin administration to pregnant females had a delayed effect on both the metabolic phenotype of the offspring in the postnatal period, and on fetuses and placentas. Leptin administration to mothers reduced offspring growth rate in the first weeks after weaning. These results are consistent with the results obtained previously, demonstrating that hyperleptinemia during pregnancy reduces the weight of the offspring during their growth after weaning (Makarova et al., 2013; Pollock et al., 2015). In this work, we have shown for the first time that leptin administration to pregnant females has an up-regulating effect on the level of IGF1 mRNA in the liver of fetuses at the end of pregnancy. IGF1 has multisystem effects on fetal development (Hellström et al., 2016), and it is possible that the programming effect of maternal leptin on postnatal metabolic traits and offspring growth is partly mediated by its influence on Igf1 expression in fetuses

The programming effect of maternal leptin was more pronounced in male offspring: only in males, administration of leptin to mothers increased fat mass, plasma concentrations of glucose, cholesterol, and triglycerides and decreased the expression of the Agrp gene in the hypothalamus and the genes for glucokinase and fatty acid synthase in the liver. Sex differences in the response to elevated maternal leptin were also observed at the prenatal stage of development: only in male fetuses, administration of leptin to mothers increased the expression of the Dnmt3a gene in the liver. DNMT3a mediates de novo methylation (Jurkowska et al., 2011) and maternal influence on fetal liver expression of this enzyme may have delayed effects on mature liver gene expression. In turn, changes in the expression of genes encoding enzymes in the liver can affect the metabolic parameters of the blood. Thus, a decrease in the expression of the glucokinase gene may be the cause of an increased blood level of glucose in males born to leptin-treated mothers, since glucokinase is a major contributor to glucose homeostasis (Massa et al., 2011), and a decrease in the expression of the Gck gene is accompanied by an increase in the level of glucose in the blood (Magnuson et al., 2003).

Despite the pronounced sex differences in metabolic characteristics and the sex-specific effect of maternal leptin on the metabolic phenotype of the offspring, the programming effect of maternal leptin on adaptation to SFD consumption did not depend on the offspring sex. Leptin administration to mothers did not pronouncedly affect the metabolic response and transcriptional changes in the liver and brown fat caused by SFD consumption, but affected the central mechanisms regulating energy intake and expenditure. In both sexes, administration of leptin to mothers doubled the expression of the Crh gene in the hypothalamus when SFD was consumed. Hypothalamic corticotrophin-releasing hormone (CRH) coordinates energy intake and expenditure with metabolic and behavioral response to stress (Richard et al., 2000). CRH in the hypothalamus has an anorexigenic effect and increases energy expenditure (Radahmadi et al., 2021). Decreased sensitivity of CRH neurons increases susceptibility to obesity in mice (Zhu et al., 2020). Since the increase in Crh gene expression was not accompanied by changes in food intake and body weight, it can be assumed that maternal leptin affected the response of hypothalamic–pituitary–adrenal axis to metabolic stress caused by SFD consumption. The nature of these influences requires additional research.

In addition, leptin administration to mothers affected the hypothalamic expression of orexigenic (Agrp) neuropeptide in males and anorexigenic (Pomc) neuropeptide in males and females. It is assumed that prenatal programming of the metabolic phenotype is mediated via epigenetic modifications of the central systems that regulate energy intake and expenditure (Dearden, Ozanne, 2015). Thus, it has been shown in laboratory models and humans that the metabolic state of mothers during pregnancy (malnutrition, overeating) affects methylation of the gene encoding proopiomelanocortin and, accordingly, its expression in the hypothalamus in the offspring (Candler et al., 2019). In rats, maternal consumption of high-calorie diet significantly increased basal CRH mRNA expression in the paraventricular nucleus of hypothalamus (Niu et al., 2019). Our results indicate that leptin may be the factor mediating maternal influences on the central regulation of energy homeostasis.

Although we found no sex-dependent programming effects of maternal leptin on adaptation to SFD eating, its sex-specific influence on liver gene expression and metabolic characteristics may promote formation of sex differences in the development of diet-induced obesity in offspring.

## Conclusion

Males differ from females in metabolic features associated with glucose and lipid metabolism, as well as adaptation to excess energy intake with a high-calorie diet. Leptin administration to pregnant female mice sex-specifically affects liver gene expression and metabolic characteristics in adult offspring. This sex-specific programming effect may be associated with sex-specific influence of maternal leptin on expression of the Dnmt3a gene in fetal liver. Regardless of sex, maternal leptin had a programming effect on the activity of the hypothalamic CRH system during adaptation to SFD consumption

## Conflict of interest

The authors declare no conflict of interest.
